# The Gut Microbiome Feelings of the Brain: A Perspective for Non-Microbiologists

**DOI:** 10.3390/microorganisms5040066

**Published:** 2017-10-12

**Authors:** Aaron Lerner, Sandra Neidhöfer, Torsten Matthias

**Affiliations:** 1 B. Rappaport School of Medicine, Technion-Israel Institute of Technology, Bat Galim, Haifa 3200003, Israel; 2 AESKU.KIPP Institute, Mikroforum Ring 2, 55234 Wendelsheim, Germany; neidhofer@aesku.com (S.N.); matthias@aesku.com (T.M.)

**Keywords:** intestine, gut, brain, axis, mechanisms, pathways, microbiome, dysbiome, autoimmunity

## Abstract

**Objectives:** To comprehensively review the scientific knowledge on the gut–brain axis. **Methods:** Various publications on the gut–brain axis, until 31 July 2017, were screened using the Medline, Google, and Cochrane Library databases. The search was performed using the following keywords: “gut-brain axis”, “gut-microbiota-brain axis”, “nutrition microbiome/microbiota”, “enteric nervous system”, “enteric glial cells/network”, “gut-brain pathways”, “microbiome immune system”, “microbiome neuroendocrine system” and “intestinal/gut/enteric neuropeptides”. Relevant articles were selected and reviewed. **Results:** Tremendous progress has been made in exploring the interactions between nutrients, the microbiome, and the intestinal, epithelium–enteric nervous, endocrine and immune systems and the brain. The basis of the gut–brain axis comprises of an array of multichannel sensing and trafficking pathways that are suggested to convey the enteric signals to the brain. These are mediated by neuroanatomy (represented by the vagal and spinal afferent neurons), the neuroendocrine–hypothalamic–pituitary–adrenal (HPA) axis (represented by the gut hormones), immune routes (represented by multiple cytokines), microbially-derived neurotransmitters, and finally the gate keepers of the intestinal and brain barriers. Their mutual and harmonious but intricate interaction is essential for human life and brain performance. However, a failure in the interaction leads to a number of inflammatory-, autoimmune-, neurodegenerative-, metabolic-, mood-, behavioral-, cognitive-, autism-spectrum-, stress- and pain-related disorders. The limited availability of information on the mechanisms, pathways and cause-and-effect relationships hinders us from translating and implementing the knowledge from the bench to the clinic. **Implications:** Further understanding of this intricate field might potentially shed light on novel preventive and therapeutic strategies to combat these disorders. Nutritional approaches, microbiome manipulations, enteric and brain barrier reinforcement and sensing and trafficking modulation might improve physical and mental health outcomes.

## 1. Introduction

For many years the brain was considered the prime organ, dominating our organs, systems, behavior and personality. The role of the central nervous system (CNS) in terms of brain–gut interaction has been considered a significant factor for multiple intestinal functions, including motility, digestion, absorption, local hormonal secretion and visceral sensitivity [1]. In fact, the CNS was regarded as the regulator of the enteric nervous system (ENS) via its two regions: the sub-mucosal and the myenteric plexuses. More so, until the beginning of the 21st century, the CNS was considered as an immune-privileged site, sealed by the blood–brain barrier, thus preventing infiltration by peripheral immune cells and mediators.

A recent advancement in the medical and scientific knowledge as well as in concepts and modern techniques has changed the unidirectional brain to gut relationship to include the peripheral enteric influence on the brain, namely the gut to brain cross talks. Nowadays, this axis is considered to be bidirectional, harmonizing the functions of these two complex organs, under physiological conditions or deregulated in pathological conditions [2]. The bidirectional communication between the intestine and the brain is regulated at a neuronal, endocrinal and immunological levels.

However, there is an additional kingdom that the medical and scientific communities have started to unravel. All the above-mentioned pathways are under the influence of the gut microbiome, together complementing the brain–gut-microbiota axis [3,4,5,6]. The gut microbiota is a key player in gut health and function. It is composed mainly of bacteria but also of archaea, viruses and protozoa, reaching roughly 10^14^, and the ratio of bacterial to host cells in human is close to 1:1. Its dimensions, composition and activities have led to its description as a “superorganism” [7]. Evolutionarily, bacteria and African genus Homo emerged roughly 3.8 billion and 2.5 million years ago, respectively, thus providing the microbes a much longer time to adapt, evolve and develop survival mechanisms, long before bugs inoculated us [8]. Along the way, an adjusted relationship between the host and microbial cells was molded over a very long co-evolutionary process. The gut microbiota is particularly interesting as it shows both amazing resilience to perturbation, and also dynamic variability and plasticity over time and body site. Due to the importance of the gut-microbiota–brain axes and the environmental effects on the enteric microbiome, the present review aims to update on the interrelationship between nutrition, and gut microbial eco-system-intestinal events and the brain. More specifically, based on the current evolving knowledge, the mechanistic pathways whereby the microbiota is influenced and influences, the gut–brain axis will be described.

## 2. Potential Intestinal Eco-Events That Affect the Microbiome

### 2.1. Nutrients, Food Additives, Bugs and Us

Many environmental factors impact the gut microbiome. Geography, lifecycle, mode of delivery, infant feeding, stress, exercise, hygiene, infections, pharmaceuticals and food are some examples [9,10,11,12,13]. Diet has emerged as one of the most relevant factors in influencing the gut microbiome. In reality, nutritional customs have a critical impact on human health, affecting an individual’s risk for various chronic diseases. The ‘westernization’ of worldwide eating and lifestyle modifications is associated with an increasing rate of cardiovascular, cancerous, metabolic and allergic diseases. Moreover, an individual’s lifestyle selection can markedly affect the progression and manifestation of autoimmune diseases [10,11,12]. In light of these outcomes, it is logical that the search for alternative therapies to combat such diseases would include inquiries into lifestyle modifications [14]. Nutrition, from as early as in utero, through the neonatal period, and up to adulthood, has a profound effect on the shape and trajectory of our intestinal microbiome. New genetic technologies and bioinformatics reveal the immense influence the enteric microbiome has on our early development, intestinal homeostasis, behaviors and susceptibility to and recovery from human diseases. When nutrients enter the human intestinal bioreactor much of human physiology is changed, including major effects on the gut microbiota composition, diversity and metabolomic product secretion.

Significant changes in the gut microbiome have been primarily associated with the intake of fiber from fruits, vegetables and other plants. In this regard, De Filippo et al., compared the gut microbiome of African to Western children [15]. Upon comparison of a vegetarian diet (low fat, low animal protein, abundant in starch, plant polysaccharides and fiber) to a Western diet (plentiful in animal protein, sugar, starch and fat, but short in fiber), relevant discrepancies were depicted in the four major phyla: *Actinobacteria* and *Bacteroidetes* increased in the African group while *Firmicutes* and *Proteobacteria* were more plentiful in the European branch of the study. Interestingly, the African children exclusively harbored short fatty acid (SCFA)-producing bacteria that use xylen, xylose and carboxymethylcellulose, thus producing four times more SCFA. SCFA was described as an anti-inflammatory at the gut levels [16]. De Filippo et al., suggested that the African children’s microbiome co-evolved with their diet to assist with energy harvest by producing higher levels of SCFA [15]. When the fecal flora of adult vegetarian/vegan subjects were compared to an omnivorous diet, the first group disclosed a lower microbial count of *Bifidobacterium*, *Bacteroides*, *Escherichia coli* and *Enterobacteriaceae* and lower pH, compared to the second group [17]. The highly enriched indigestible carbohydrate and fiber diet of the vegetarian/vegan subjects is the origin of the higher SCFA content, resulting in the lower stool pH. It is well known that dietary fibers are related to the high production of SCFAs by the gut microbiota and, in turn, with the induction of immune tolerance [18].

Despite our growing knowledge, less is known about the interplay of nutrients and gut microbiota in immune-mediated diseases. Dietary milk, carbohydrates, fats, protein, fiber, fruit, vegetables, animal proteins, sodium chloride and aluminum [19] were investigated as potential inducing factors in Crohn’s disease [20]. Cow milk, fruit and berry juices, and n3-polyunsaturated fatty acids were explored in type one diabetes. Even the incidence of multiple sclerosis was positively associated with the consumption of milk, animal fat and meat, total energy intake and resulting obesity [21].

Contrary to disease induction, multiple nutrients were suggested as acting as anti-inflammatory agents, and thus might have protective or preventive effects. These include, at least in rheumatoid arthritis, fish and primrose oils, black cumin, fenugreek, licorice, coriander, tomato, carrot, sweet potato, broccoli, green tea, rosemary, hazelnut, walnut, wheat germ and dates. In celiac disease (CD), long chain ω-3 fatty acids, plant flavonoids and carotenoids appeared to modulate oxidative stress, inflammatory mediators and gene expression. More so, phytonutrients such as lycopene, quercitine, vitamin C and tyrosol were suggested to protect against the cytotoxic effects of gliadin. Nevertheless, the majority of investigations have been equivocal or circumstantial and do not yet validate any of these nutrients as causal factors [22]. It should be noted that those nutritional epidemiological studies have not integrated microbiome stool analysis, therefore the role played by a specific nutrient on the microbe’s composition and function is far from being elucidated.

It seems that the dietary exposome is far from clarifying the microbiome behavior and the human reactome.

In addition to food and nutrients, the industrial food processing additives also affect enteric eco-events. Glucose, salt, emulsifiers, organic solvents, gluten, microbial transglutaminase, and nanoparticles, which are increasingly used in industrial food processing, impact microbiota composition. They are also considered to breach the enteric tight junction (TJ) integrity and are potential inducers of the autoimmune cascade [10]. More so, microbial transglutaminase (mTg) that functionally imitates the tissue transglutaminase (tTg) (the autoantigen of CD), was lately shown to be immunogenic in celiac disease patients [12,23].

A distinctive place should be dedicated to gluten, a universally consumed nutrient. Considering the analogous increase in world-wide gluten intake and chronic, non-infectious diseases incidences, it is proposed that gluten might have biologically detrimental effects [24]. In fact, gluten has multiple side effects, affecting human health, characterized by gluten dependent digestive and extra-digestive signs and complaints that may be arbitrated by immunological reactions and primed by gastrointestinal inadequacy. In the enteric lumen, it affects the microbiome composition and diversity and enhances intestinal permeability. Gluten is immunogenic and cytotoxic, pro-inflammatory and drives the innate and adaptive immune systems. On the cellular level it augments apoptosis, decreases viability and differentiation and influences nucleic acid and glycoprotein synthesis. It has many systemic effects as a pro-inflammatory, and affects epigenetic pathways. On therapeutic level, a gluten-free diet, in certain non-celiac autoimmune diseases patients (type one diabetes, rheumatoid arthritis, multiple sclerosis, psoriasis, autoimmune hepatitis and thyroiditis) may be helpful to reduce gluten’s disadvantageous effects [24]. It appears that early diagnosis of CD, on gluten withdrawal, is protective for other associated autoimmune diseases [25], an effect not seen on late CD diagnosis [26]. Most recently, even in the veterinarian world, a gluten-free diet improved the epileptoid cramping syndrome in Border Terrier dogs [27].

Finally, in a more optimistic approach, based on the influences of the Western lifestyle on adiposity, glucose metabolism, oxidative stress and inflammation, bacterial strains and their metabolic products that are beneficial under this lifestyle were selected as the most promising probiotic isolates [28]. It seems that we are beginning to unravel the importance of the microbial key components that might hinder the evolution of human chronic diseases.

In summary, depleted microbial biodiversity of the gut microbiota in people consuming a Western diet is linked to increasing incidence of obesity, coronary vascular disease, stroke, metabolic syndrome, autoimmune diseases as well as an increased risk of malignancies. Improving dietary habits towards a long-term consumption of a “healthy” versus an “unhealthy” diet, will impact substantially the microbiota/dysbiota balance. The popular sentence, “we will be what we eat or what we were fed” [29] should include the microbiome as a gate keeper between food and mankind’s health. The association between this agrarian-based diet with specific bacterial taxa, a surge in microbial richness, at the taxonomic and genetic levels, and improved health compared to Western diets has been consistently established. In view of the food effects, it makes sense that the quest for nutritional therapies to abate the initiation and progression of chronic diseases would include explorations into more holistic lifestyle changes.

After the description of the dietary influence on the microbiota profile, the following will expend on the microbial reactome, setting the stage for the gut-microbial–brain axis.

### 2.2. Microbial Metabolome as Mobilome

Although food affects the compound and diversity of the intestinal microbiome, more significant are its impacts on the metabolome. The enteric ecosystem, overloaded with microorganisms and compacted immune system cells can be viewed as an isolated compartment on its own. Under dysbiotic states, however, the microbiome/dysbiome equilibrium is changed and results in an abnormal interaction between the bugs and us. Some enteric microbiome dwellers have been associated with specific chronic human conditions including autoimmune diseases and food allergies [30,31]. Changing a single microbial species and/or the entire commensal community can modify the outcome of a specific autoimmune disease due to the imbalance of detrimental/protective immune responses [32]. A list of specific bacterial species, related to defined animal models of autoimmune diseases and their functions, in relation to disease development, was most recently reported [30]. However, no phenotype–microbial relationship or cause-and-effect relationship, to our ample knowledge, was established for any of those chronic conditions.

The gut microbiota produce endless and constantly changing metabolites that impact host physiology and susceptibility to disease, however, the causative molecular events remain largely unknown. Nutrition-induced alterations in the composition of the enteric microbiota can modulate the recruitment of regulatory versus effector immune responses at the intestinal level and ameliorate the health outcome. Prebiotics, probiotics and dietary fiber are the main means for prophylactic and therapeutic intervention against intestinal inflammation [33]. Most recently, the relationships between diet, the microbiota, metabolomics, and gene function was further clarified on an animal model and in CD [16,34]. It was shown that bacterial colonization modulates global histone acetylation and methylation in various host tissues in a diet-dependent manner: intake of a “Western-type” diet deprives many of the microbiota-dependent chromatin changes that occur in a polysaccharide-rich diet. Supplementation of germ-free mice with SCFAs, a major metabolite of gut microbial fermentation, was sufficient to renew chromatin modification status and transcriptional reactions associated with colonization [34]. In fact, the most studied metabolic products, that have beneficial effects, are SCFAs. SCFA-mediated signaling pathways are vital for enteric bacterial communication with the host. They regulate immune functions, intestinal hormone production, lipogenesis and many more luminal and systemic influences [16]. Interestingly, butyrate promotes colonic health and helps to prevent cancer [35].

Acetate, propionate, butyrate, and pentanoate, with two, three, four, and five carbon atoms, respectively, are SCFAs, largely made by bacterial fermentation of non-digestible polysaccharides like starches and fibers in the colonic lumen. After being absorbed by the gross intestine epithelium, where the preferred fuel source of colonocytes is butyrate, they enter the bloodstream through the portal vein of the host and/or the distal colon. Then, they are distributed to peripheral organs where they are taken up, metabolized and used in multiple cellular responses [16]. Contrary to the variability in the loss of diversity of the microbiome repertoire in autoimmune diseases, less is known about the source of the luminal metabolites. SCFA production is greatly related to food, but the specific microbial species rate of SCFA output is yet unknown. A metabolic signature in the lumen and stools of specific and total SCFAs, in CD, for example was described [16]. However, a long-term gluten-free diet did not completely restore the microbiome in the metabolome of CD children [36]. One of the actions of the luminal SCFAs is the increase of mucosal immune tolerance by the activation of G-protein-coupled receptors and the subsequent activation of T regulatory cells [37].

Despite the proposal that probiotics (e.g., Lactobacillus and Bifidobacterium) may alter the metabolism in the colon by enhancing the production of SCFAs, we are far from “rebiosis” or the answer to the question: how can bacterial diversity and functionality be restored in dysbiotic or in pathobiotic circumstances?

However, SCFAs are not the only metabolic products. The list of diet-dependent, microbial-originated metabolic products that improve or deteriorate human health is constantly increasing [38]. Food rich in phosphatidylcholine is a main source of choline. Catabolism of choline by the gut microbiome induces the formation of gas and trimethylamine, which is metabolized by the liver into trimethylamine oxide, a small molecule that is firmly related to the increased risk for coronary vascular diseases [39]. Red meat rich l-carnitine also induces trimethylamine oxide production [40]. The importance of the metabolome in predicting host dysbiosis was recently evaluated [41]. Using machine learning techniques and computational predictions, the authors showed that the aggregates predicted the community enzyme function profile and that modeled metabolomes of a microbiota are more predictive of dysbiosis than either observed microbiome community composition or predicted enzyme function repertoires. Table 1 summarizes some of the gut microbiotic beneficial and harmful metabolites in physiological and pathological conditions, respectively [42,43].

Overall, the metabolomic profile has deep implications for comprehending the complex interactions between diets, gut microbiota and host health. This brings a potential promise of nutritional manipulations of the gut microbiome and its metabolites as a way to improve health and treat diseases. Nutrigenetics, nutrigenomics, personal diets or purified metabolomic compounds are a few of the future therapeutic strategies to improve nutrient, metabolome, and gut performance for the benefits of mankind [44].

### 2.3. Post-Translational Modification of Naïve Proteins

Post-translational modification of proteins (PTMP) dominates numerous pathways related to cellular metabolism, representing a key regulator of autoimmunity and potentially of allergy [21,30].

Bacteria have an astounding capability for accommodation and survival strategies, comprising different utterances of the transcriptome and proteome, disparities in growth rate, and withstanding extreme conditions. PTMP contributes significantly to this adaptability and microbial life cycle modifications. Additionally, bacterial PTMP represents a substantial importance to the host. Their enzymatic capacities to transform the naïve/self or non-self-peptides to autoimmunogenic or allergenic forms, is extensive. A large list of enzymes synthesized by dysbiotic populations, capable of PTMP, was published lately [30].

A known example of PTMP is the tissue transglutaminase (tTg) in CD or peptidylarginine deiminases in rheumatic arthritis, where deamidation/crosslinking of gliadin or citrullination occur, respectively [46,47]. In CD, the autoantigen is tTg, capable of deamidating or cross-linking gliadin [23]. This PTMP takes place below the epithelium, where neo-epitopes of gliadin docked on the tTg are created, provoking anti-tTg or anti neo-epitope tTg autoantibodies synthesis. Those are well established biomarkers of CD [48]. Recently, a family member of tTg, the microbial Tg, abundantly used by the processed food industry, was described to be a potent inducer of specific antibodies in CD patients [12]. More so, the same food ingredient has been suggested as a new environmental trigger and potential inducer of CD [12,23,49]. Very recently, only CD patients, and not controls, were shown to raise specific antibodies against the cross-linked complex between the microbial Tg and the gliadin [12]. Moreover, PTMP is an important intestinal luminal event that potentially contributes to the extraintestinal phenotype development in CD [21]. In rheumatoid arthritis, citrullination of peptides, by the bacterial enzyme peptidylarginine deiminases is a prototype of PTMP. Most recently, we put forward the hypothesis that the cerebral tTg or potentially the microbial Tg might be involved in neurodegenerative diseases [50,51]. Being a universal protein crosslinker and translational modifier of peptides, the tTg and/or the microbial Tg can crosslink various peptides, to be deposited in the brain, in a folded or misfolded configuration, thus imitating neurodegenerative processes [52]. The intestinal microbiota, dysbiota, pathobiome, probiotics and processed food contribute to the luminal bacterial origin Tg daily cargo [50]. It is hypothesized that those bacterial enzymes potentially steer neurodegenerative and neuroinflammatory diseases via intestinal luminal events. By crosslinking naïve proteins, the enzyme can potentially create neo-epitopes that are not only immunogenic but may also be pathogenic, activating some pathological pathways in the cascade of chronic CNS disease induction. The detrimental activities of the bacterial Tg may represent a new mechanism in the gut–microbiome–brain axis and might open novel therapeutic strategies to combat those degenerative diseases. In fact, tTg is a disease-modifying factor in neurodegenerative diseases, because tTg might enzymatically stabilize aberrant aggregates of proteins involved in those conditions. The enzyme contributes to the aggregation of huntingtin protein, insoluble neurofibrillary tangles and β-amyloid plaques, or α-synuclein in Huntington’s disease, in Alzheimer’s disease and in Parkinson’s disease, respectively. Tg is additionally involved in neurotransmitter release states like the botulinum and tetanus neurotoxins activities [53].

Concerning allergies, tTg is involved in wheat allergy [54], PTMP participates in mugwort pollen allergy [55] and delay type hypersensitivity [56]. Neo-epitope formation by PTMP is shared in autoimmunity as neo-immunogens, as well as in allergies, as neo-allergens.

Returning to the microbial Tg, it has been proposed that the whole family of microbial Tgs are proteases and that the eukaryotic Tgs have evolved from an ancestral protease [57]. Taking into account that proteases and anti-proteases are drivers of PTMP and can potentially breach tight junction integrity [45,58], the microbial Tg becomes a potential enhancer of intestinal permeability, thus setting the stage for the next section.

### 2.4. Increased Intestinal Permeability: The Leaky Gut

The intestinal barrier illustrates a vast surface of 400 m^2^, where billions of microbes confront the vastest immune apparatus in the human body. Humans support a very complex microbial ecosystem peacefully coexisting with the microbiotic cargo, which tightly interacts with the underlying immune systems. It has been proposed that the human genome cannot support all duties and functions required to survive, since the gut microbiota is crucial to maintaining health and protecting against the pathobiome and numerous diseases [59,60]. Our symbiotic microbiome endows multiple metabolic capacities that the mammalian genome-dependent metabolome lacks. One of the microbiome-dependent pivotal functions is to maintain the functional integrity of the intestinal barrier [45,60,61,62]. The gut barrier is composed of the mucus layer, epithelial layer and the underlying lamina propria. The tight junction (TJ) machinery is situated between the enterocytes, connecting the gut epithelial cells and regulating the paracellular permeability. It prevents the loss of water, electrolytes and small molecular nutrients, and the entry of antigens, toxins and microorganisms inside our body. Such opposing functions are much regulated, microbiome-dependent, extremely orchestrated and evolutionarily conserved under normal conditions. Its key role in avoiding inflammatory responses to the microbiota is heavily dependent on the fine-tuned mucosal and systemic immune networks for microbial recognition and tolerance induction. Loss of this barrier may result in enhanced epithelial permeability to gut microbiota or additional luminal components, which may lead to the phenomenon of molecular mimicry, a well-known pathway of autoimmunogenesis.

Numerous and various categories of potential TJ disruptors exist. Some of them are listed in Table 2. In fact, multiple human conditions have been associated with dysbiotic alterations or reductions of the microbiota’s diversity, including cancer, inflammatory bowel diseases, food allergies and other atopic conditions, critical illness, irritable bowel syndrome, non-celiac or celiac gluten sensitivity, and metabolic diseases such as diabetes mellitus type two and obesity, cardiovascular, non-alcoholic fatty liver, or non-alcoholic steatohepatitis diseases and neuropathologies [45,60,61,62]. Additionally, TJ functional impairment is a primary defect in autoimmune diseases [63,64,65]. Intestinal permeability is increased in many of them: ulcerative colitis, Crohn’s disease, CD, inflammatory joint disease, ankylosing spondylitis, juvenile onset arthritis, psoriatic arthritis, diabetes mellitus type one and primary biliary cirrhosis. In fact, the loss of the protective capacity of the mucosal barriers that interact with the outside world is necessary for autoimmunity, allergy, inflammatory, metabolic and some cancer diseases to develop [10,63,64,65,66]. Since balanced homeostasis is the physiological rule and since many TJ distractors exist, counteracting environmental factors that protect or improve TJ functions should operate. Table 3 summarizes the factors that protect intestinal permeability and might present potential new therapeutic strategies.

**Figure 1:**
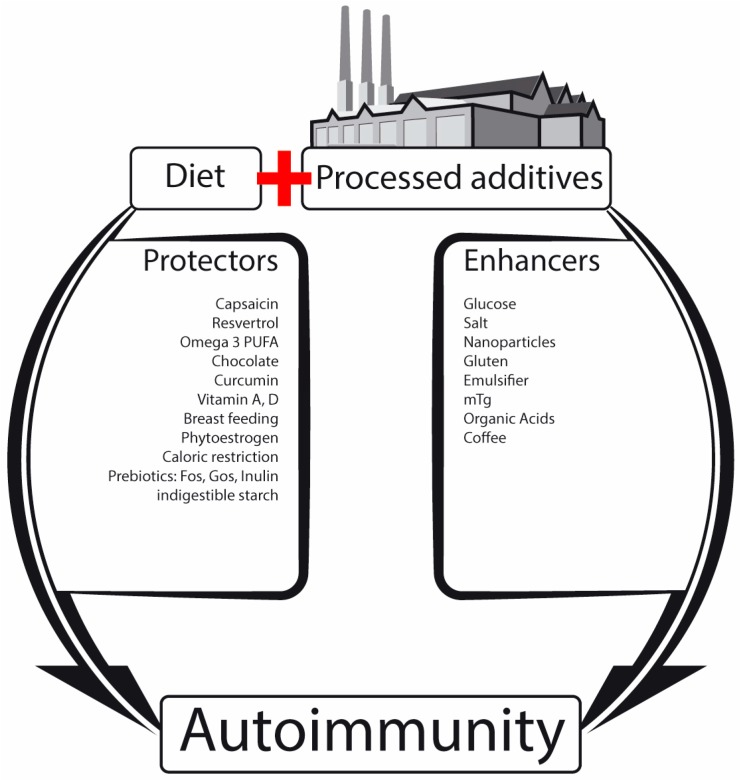
Schematic illustration of the factors that are associated with increasing (enhancers) or decreasing (protectors) of intestinal permeability at the TJ level. The leaky gut might initiate the autoimmune cascade. (Adapted from references [10,12,20,36,45,46,49,50,51,52,53,54,56,57,58]).

Taking together, TJ dysfunction, frequently called ‘leaky gut’, and its pathophysiological consequences on the pathogenesis of chronic human diseases is constantly unraveled, but many aspects remain unclear. Is it a cause, consequence or co-evolutional phenomenon that the gut ecosystems drive [2]? Accumulating information suggests that intestinal luminal eco-events, whereof the microbiome is a major one, might alter the regulatory mechanisms of the TJ. This results in a leaky gut, thus shattering the balance between tolerance and immunity to non-self-antigens. Metabolomic products, microbial constituents, transformed neo-epitope peptides, immunogenic/pro-inflammatory molecules, toxins, allergens, carcinogens, drugs, pathobionts and nutritional products can potentially be transported systemically, reaching remote organs, including the brain [2]. Figure 1 illustrates schematically the factors that are associated with increasing (enhancers) or decreasing (protectors) of intestinal permeability at the TJ level. Breached TJ integrity might represent a crucial step in the gut–brain hinge.

## 3. The Enteric Systems That Receive and Transmit Messages to the Brain

### 3.1. The Intestinal Glial Neuronal Bouncer (Microglial Network)

After describing various topics such as the nutritional factors that affect the intestinal microbiome, the metabolomic consequences, the place of neo-peptide formation by PTMP, and leaky gut syndrome, it is time to tie together the enteric eco-events and to look for the enteric receptive systems for those luminal and mucosal messages that connect the gut to the brain. The most logical local detecting set is the intestinal neuronal networks, serving as a sensing system, through messaging pathways, distribution and delivery arrays to the CNS. This is the correct place to state that the gut–brain circuitry is much more complicated, since the enteric events exert their effects not only through the nervous network, but also channeled through the enteric endocrine, immune and metabolic systems [5,6].

The neurons of the enteric neuronal system contain two types of interconnected ganglia: myenteric (Auerbach’s) and sub-mucosal (Meissner’s) plexuses. Myenteric plexuses are located between the inner and outer layers of the muscularis externa, while sub-mucosal plexuses are located in the submucosa. Those two intestinal plexuses and their tight connections to the autonomic nervous system and the vagus nerve were extensively evaluated and described [3,4,5,6,60,71,72,73,74,75,76,77,78,79] and multiple schematic presentations were provided [4,5,77,78,80,81,82,83]. The present review aims to expand and update on the third neural anatomic pathway, namely, the gut glial network, where new interesting information is continuously accumulating on its development [84,85,86], regulation [85,86] and roles and functions [80,81,82,83,87,88]. Just for anatomical/histological orientations, there are several-fold more glial cells than neurons in the enteric neuronal systems and they dwell in the enteric plexuses (intra-ganglionic glial), along the nerves in the enteric circular muscle layer (intra-muscular glial) and in the lamina propria beneath the epithelium in contact with the basement membrane, sub-epithelial myofibroblasts and lymphatic vessels (mucosal glial) [82,86]. They form a continuous network in the lamina propria from the base of crypts, up to the crypt openings [80]. In fact, these cells resemble astrocytes of the brain. It should be emphasized that despite the mucosal partners—such as the unilayered epithelium and the basal membrane—local immune and enteroendocrine cells, blood vessels and lymphatics, various nutritional/bacterial components/metabolites and other molecules that find their inter-/intracellular pathways penetrate the epithelium. They are situated at a very strategic cross-road to integrate intercellular signaling and coordinate the afferent information toward the central nervous system [82,89]. Similar to interactions such as luminal and mucosal intermingling, modulation and cross-talking eco-events, the neurons and the glial cells modulate the intestinal epithelial barrier functions. Moreover, the glial cell’s homeostasis is regulated by the microbiome [85,86]. The increasing knowledge on the topic has given rise to a new concept of a digestive “neuronal–glial–epithelial unit” akin to the brain neuronal–glial–endothelial network [83]. The recent development of this domain even introduced a new window to the ENS, represented as a new scientific and medical sub-specialty, namely neurogastroenterology [90]. The next section will concentrate on the role of the enteric microglial network in the microbiota–TJ–gut–brain axis.

### 3.2. The Enteric Glial Roles

#### 3.2.1. The Intestinal Local Roles

It appears that most intestinal functions are regulated by the ENS and, due to its key role, abnormalities in its formation or functions cause several morbid or life-threatening human diseases. It extends all along the gastrointestinal tract, and very intricately and uniquely orchestrates gastrointestinal behavior, independent of the brain nervous compartment. Its functional integrity is pivotal for life and dysfunction is linked to various congenital or acquired digestive disorders [84,91,92]. More appropriate to the present topics are the central nervous system conditions that have lately been associated with its dysfunction: autism spectrum disorder, amyotrophic lateral sclerosis, transmissible spongiform encephalopathies, and the Parkinson and Alzheimer diseases [92,93]. Many of them present gastrointestinal comorbidity [92].

The enteric glial network is an integral part of the intestinal nervous system and the following section will retrench its local enteric roles.

Multiple functions were allocated to the intestinal glial network:

1. Maintenance of barrier functions [80,81,82,83,87,88].
2. Protect enteric functions by glial cell mediators or by the modulation of neurotransmission and secretion in the GI tract [87].
3. Provide trophic and cytoprotective functions towards enteric neurons [87,88].
4. By possessing receptors for various enteric neurotransmitters, they are activated by synaptic transmission e.g., ATP release from stimulated or damaged neurons or from the site of tissue trauma, infections, immune insult or inflammation [87].
5. The glial cells respond to and produce cytokines and chemokines (IL-1 receptor, IL-1, IL-6, monocyte chemotactic protein1) that impact local events [87].
6. Regulate neuronal activity [82,88,93]. They ‘listen’ to neuronal conversations.
7. Protect local tissue integrity [87].
8. Control GI motility [80,88].
9. Regulate mucosal secretion [82,87].
10. Regulate mucosal immunity [87,88].
11. Active as progenitor cells [93].
12. Protect, support and maintain the mucosal neural network [82,87,88,93,94].
13. Constrain microbiota composition towards increased anti-inflammatory and decreased pro-inflammatory bacterial lineages [95].
14. Modulate epithelial cell proliferation, differentiation and healing [96,97].
15. Defend intestinal mucosa against pathogen invasion [88,98,99].

Due to its pivotal functions in the intestinal tract, when the enteric glial cell dysfunctions occur, various gastrointestinal diseases appear. Motility disorders [100] such as chronic idiopathic intestinal pseudo-obstruction [80,91], post-operative ileus in mice [80], chronic constipation [94,101], or fulminant jejunoileitis and infectious gastroenteritis [80,94], gastroschisis [102], and inflammatory bowel disease, such as Crohn’s or ulcerative colitis [80,94,103], intestinal inflammation [104] and irritable bowel syndrome [105] are associated with intestinal glial cell depletion, malfunction or failure. Notably, in most of those pathologies it remains unclear whether cause-and-effect relationships exist or the glial network abnormalities are merely the consequence of those conditions. Glial cell depletion occurs during the aging process, but, as mentioned above, the question, “if enteric neurogliopathy precedes or follows aging”, is still debatable [94].

Finally, a holistic view should be applied, instead of delineating each puzzle’s compartment of the human gut. In between the microbiome and the local glial network, the intestinal epithelium and the mucosal immune systems plugged a stake. Both serve as intermediaries between the gut microbiota and mucosal glial apparatus [85,88,106,107,108]. Bugs, gut and glial are not only in anatomical proximity, but they also influence and regulate each other and are interconnected for mutual homeostasis [86].

It seems that “the microbiota keeps enteric glial cells on the move” [109]. Most recently, the “gut connectome: making sense of what you eat” was coined for the enteric neuronal ensemble, emphasizing its direct and functional cross-talks with the other enteric mucosal and luminal compartments [110]. It seems that during mankind’s evolution a ‘brain’ was evolved in the gut, represented by the local intermingled nervous network [111].

#### 3.2.2. Role of Mucosal Glial Cells in Brain Disorders

As mentioned above, the mucosal glial network is associated with multiple gastrointestinal pathologies. Although logically counterintuitive, the enteric glial network that dwells in the gut wall, is involved, however, in a constantly growing list of brain disorders [80]. The most explored one for the relationship between the enteric nervous network and the brain is Parkinson disease (PD) [80,92,93,112,113]. Multiple gastrointestinal manifestations were described: constipation, defecatory dysfunction, drooling, dry mouth, dysphagia, nausea, vomiting and gastroparesis. Some of the symptoms preceded motor manifestations [112,114]. Interestingly, several features support the hypothesis that the PD process spreads in a caudal-cephalic direction from the gut to the brain, with enteric symptoms preceding motor abnormalities, preliminary accretion of abnormal α synuclein-containing Lewy inclusions in the ENS and in the attached glial cells with a rostrocaudal gradient, decreasing from the upper to the lower intestine [112,113,114,115]. Pathophysiologically, Braak’s hypothesis is currently the most accepted one [116]. It states that sporadic PD is caused by a pathogen (virus or bacteria) or their byproducts, which, after entering via the nasal cavity, are swallowed, and further spread to the gut, thereby, initiating aggregation of—α synuclein in the nose and gastrointestinal tract. These aggregates spread toward the brain via the olfactory bulb and the vagus nerve, eventually arriving to the substantia nigra [116,117]. Enteric glial dysfunction, occurring in PD, is associated with the patients’ gastrointestinal dysfunction and represents a new player in PD progression [118]. Recently, a functional aspect was allocated to the enteric glial, where activation presented by glial fibrillary acidic protein expression and phosphorylation was demonstrated in PD [119]. Notably, there is a nutritional therapeutic aspect to the gut–brain spreading in PD. Since intestinal events including inflammation are considered silent early drivers of PD pathogenesis [120], and early diagnosis, better prevention and targeted management at the initiation stage is encouraged in PD [115], several food-based therapies were suggested [121]. Flavonoids, black or green tea extracts, CP1 food supplement, and dietary plant lectins are some of them [122,123,124,125]. However, the topic of non-digestible carbohydrates represents an oxymoron. On one hand, fibers are used to alleviate constipation in PD patients and on the other hand, the PD-derived dysbiota induced enhanced motor dysfunction, most probably via short chain fatty acids and competent microglia in a PD animal model, suggesting a beneficial effect of a low carb diet [126]. Many more brain affecting disorders, where the intestinal microbiome and enteric nervous networks are actively involved, are described and are summarized in Table 4.

#### 3.2.3. Potential Enteric Neuron and Glial Cell Sensing Capacities

The enteric glial network not only provides structural and nutritional support for the enteric neurons, but influences, activates or modulates close non-neuronal cells such as enterocytes, immunocompetent and enteroendocrine cells. Due to its proximity to microbes and other foreign luminal constituents, including nutrients, it has a pivotal role in protecting the self against non-self-antigens. In addition to its preventing capacity in participating in epithelial defending functions, it is crucial for the careful modulation of the inflammatory response in case of local dysfunction [88,108]. The question arises as to how the enteric nervous system and glial cells sense the foreign antigens, the luminal microbes, nutritional components or any enteric eco-events and transmit the signals locally and cephalically.

Several mechanisms have been proposed for the microbiota communication with the enteric neurons or glial cells [170]: (1) The toll-like receptors that the enteral glial and neuron express, which represent agonists to prokaryotic components such as LPS, viruses and nucleic acids; (2) The intrinsic primary afferent neurons with their numerous axonal processes extending into the intestinal mucosa. They respond to changes in luminal chemistry, mechanical distortion of the mucosa and their processes, thus capable of sensing microbial and other luminal components [171]; (3) Bacterial toxins such as cholera, *E. coli* heat labile and *Clostridium difficile* toxins can, directly or indirectly, impact enteric neurons to stimulate secreto-motor reflexes that induce human morbidity; (4) Microbial polysaccharide and prokaryotic microvesicles can influence enteric neuronal functions; (5) Engagement of adjacent mucosal enterochromaffin and enteroendocrine to secret active endocrine or neuro-mediators, such as serotonin, peptide YY, cholecystokinin and glucagon-like peptide one, thus distributing the messages locally or systemically; (6) Microbially-derived neurochemicals (gamma-aminobutyric acid, norepinephrine, serotonin, dopamine and acetylcholine) can potentially reach the enteric neuron/glial network, activating the corresponding receptors [172,173]; (7) The “body bacterial bioactive factories”, metabolites, which act as effector molecules—trimethylamine, bile acids, phenol and phenol derivatives, indole, vitamins B12 and K and SCFA—sensed by the sub-epithelial dendritic cells and the neuronal processes [174]; (8) Finally, the molecular mimicry pathway should be mentioned, explaining commonalities of structures between bugs and us. It may involve amino acid sequences, microRNAs or pathogenic or salutogenic proteinomic effects [138]. The bacterial capability to secrete effector proteins that mimic eukaryotic epigenetic enzymes, and regulators to utilize infected cells in their benefit, are an additional aspect of the mimicry [174]. Recently, a new pathway was reported where a bacterial amyloid functions as a trigger to induce α-synuclein accumulation through cross-seeding and priming the innate immune system, in animal models [175].

#### 3.2.4. Potential Pathways of Neuronal/Glial Caudal–Cephalic Signal Trafficking to the Brain

The research field of exploring the cross talk between the intestinal events, the enteric and glial nervous network and the pathway used to transmit the signals to the brain is growing continuously. The question of how nutrients and the gut microbiota affect the human brain has become a key research priority. For the time being, the majority of studies have been performed on animal models, while human studies are lacking. Several avenues have been described or suggested to convey the intestinal dynamic information centrally [5,6,156,174,176,177,178,179].

##### Anatomical Pathways (Vagal and Spinal Afferent Neurons)

Two neuroanatomical routes are known to deliver the signals from the intestine to the brain. The first is the autonomic nervous system and the vagus nerve, and the second is the ENS, including the enteroglial cells and the autonomic nervous system and vagal nerve in the spinal cord. The gut luminal contents and events and the mucosal constituents create the signals to be transmitted by four hierarchic integrative levels cephalically. The first are the enteric neural networks that include the glial cells, myenteric and submucosal ganglia. The second level is the prevertebral ganglia that regulates peripheral visceral reflex responses. The third is the spinal cord (T5-L2 sympathetic nerves, S2-S4 parasympathetic ones) of the autonomic nervous system and the brain stem nucleus tractus solitarius and the dorsal motor nucleus of the vagus nerve, which receive the afferent fibers of the vagal nerve. The fourth level are the higher interconnected brain centers, such as the basal ganglia and brainstem nuclei, spreading to the thalamus, lobus limbicus and insular vortex [6].

##### Neuroendocrine–Hypothalamic–Pituitary–Adrenal (HPA) Axis (Gut Hormones)

The intestinal antigenic cargo with its macrobiome, food and other ingredients and compounds impacts and regulates the HPA axis. The macrobiome is essential for neuroendocrine maturation and response. Stress induced corticosterone and adrenocorticotropic hormone in germ-free mice, can be partially reversed by fecal microbial transplant and completely reversed by mono-association of *Bifidobacterium infantis* [180]. Brain-derived neurotrophic factor and the 2A subtype of *N*-methyl-d-aspartic acid receptor expressions and 5-HT1a receptors in the cortex and hippocampus are microbiota dependent, as shown in germ-free mice [180,181]. Those receptors induce expression of the corticotropin-releasing hormone of the hypothalamus, thus impacting the HPA axis functions. The vicinity between the intestinal microbiota and the enteroendocrine cells potentiate endocrine activity much more strongly, amounting to more than 20 various gut hormones. It is emerging that these gut peptides (cholecystokinin, ghrelin, PYY, etc.) communicate between the microbes and the host, including the brain, by endocrine routes or by afferent neurons or the vagus nerve [176,177].

##### Immune Routes

Enteric immune system development, maturation and activities are heavily microbiome-dependent. A major route that the microbiome/dysbiome uses to communicate with the host is through the TLRs, which are an integral part of the local innate immune system. These pattern recognition receptors are shared between the enterocytes, immune cells and neurons, thus connecting the intestinal bacteria/virus, epithelium and innate immune system and nervous system. The resulting cytokine release can spread locally, but also via the blood, to reach the brain receptors and activate the HPA axis. Additionally, microbial constituents or metabolites can pass the epithelial monolayer reaching remote cells/organs including neurons and the brain [182]. In fact, as a proof of concept, data are accumulating on various metabolites circulating in mammalian blood that originated from the gut microbiota [183]. An additional immune route involving the modulation of peptide hormone signaling by gut-bacteria-derived peptide-like antigenic proteins, which can also act directly on peptide receptors, was recently suggested [184]. The role of the gut microbiota in host appetite control, impacting bacterial growth and affecting animal feeding behavior can represent a new gut–brain pathway.

##### Microbial Derived Signaling Neurotransmitters

Neuropeptides are essential mediators operating inside the nervous systems, between neurons and other cells [176,177]. They are versatile messengers in the endocrine, nervous and immune cells, thus transcending multiple boundaries. Many of those neuropeptides have shared membrane receptors with the gut hormones, thus operating in the same or similar biological activities. Multiple essential neurotransmitters are generated by the enteric microbiota. Gamma amino acid, butyric acid, dopamine, 5-HT, SCFAs, and indole are some of the examples. By direct routes or indirectly, through the gut mucosal system and its local immune system, microbial factors, cytokines, and gut hormones find their ways to the brain, thus impacting cognition, emotion, mood, stress resilience and recovery, appetite and metabolic balance and interoception and pain [177]. Another example is the PYY secreted by the mucosal L cells and through its Y1, 2, Y4, five receptors affect food consumption, energy homeostasis, emotions, cognition, mood and stress resilience [177]. Another mechanism connecting the gut microbiome to brain performance is the tryptophan metabolism with its dual emphasis on the regulation of serotonin and the kynurenine pathway. As shown by studies drawn from neurogastroenterology, the microbiota-modulated tryptophan metabolism and downstream serotonin, kynurenic and quinolinic acids, affect brain functions and behavior [178,179]. Finally, microbial enzymes can produce various virulent factors such as neurotoxins, resulting in *D*-lactate or ammonia delivery, further changing brain conduct [185].

It should be stressed that until now, a cause-and-effect relationship between committed neuropeptide functions and brain effects is still lacking. Most of the studies mainly establish a circumstantial relationship.

##### The Enteric and Brain Barrier Dams

The intestinal tight junction and blood–brain barriers are indispensable for human life and survival. However, many factors can breach tight junction integrity (see Table 2), including stress, thus allowing microbial metabolites or constituents, cytokines, toxins, allergens, carcinogens, or food additives [10,11] to enter the blood circulation. The ensuing TLR stimulation and the induced pro-inflammatory cytokines can directly influence the brain and its performances [6,185].

## 5. Conclusions

The brain–gut connection has gained awareness as a major contributor to human health, but the gut–brain axis, that is essential for daily life and contributes to human diseases, merits equal attention. Many reviews have focused on the top-down, brain to gut axis, however, the present review expands and updates from the bottom-up, namely, the gut to brain axis. This entails multiple environmental factors, gut eco-events and the two major players, nutrients and the second brain, the microbiome. The combined notion of nutrition, microbiota, mucosal, immune, endocrine, neuronal and brain circuitries are too complicated, but contain the pure truth.

**Figure 2:**
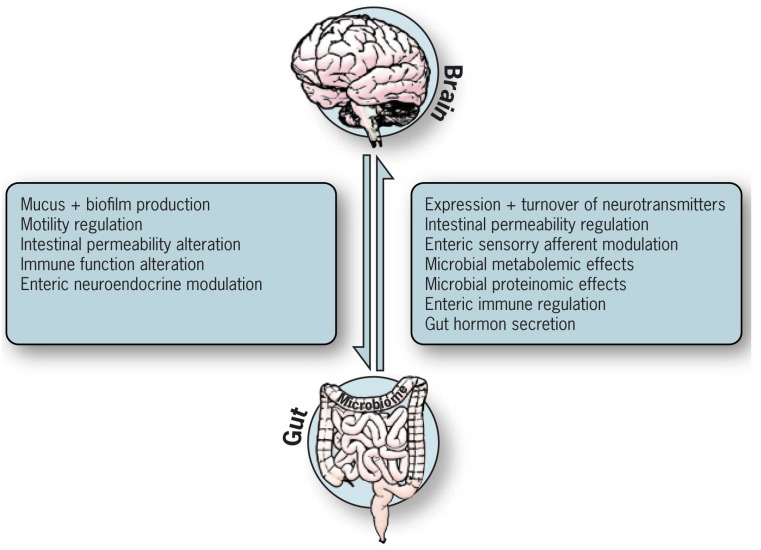
Gut–brain axis: bidirectional pathways impacting each other.

In reality, the two opposite directions refer to a bidirectional communication that mutually affects and depends on the other, as shown in Figure 2. It engulfs multiple intricate systems that were shaped during human evolution to maintain homeostasis and protect the body against detrimental factors, establishing symbiotic relations between bugs and us. Several routes are suggested to deliver the informatics knowledge from the intestinal tract to the brain: neuroanatomical, neuroendocrine, immune, macrobiotic and the gut and brain barriers pathways. Afferent vagus routes play an essential role in bringing the lower signals up to the brain. The balanced functioning of the gut–brain axis depends on normal functional activity of the vagal nerve. The present review reflects a non-infectious, gastroenterological view, and as such, concentrates more on the enteric eco-events than on the very complicated central nervous system, which is a never-ending labyrinth.

It should be stressed that the above information originated mainly from animal, in vitro and ex vivo studies, as human studies have been inadequately explored thus far. The interconnections, the mutual pathways and the exact mechanisms are more discretional and associative and the causality is waiting for future exploration.

## Figures and Tables

**Table 1:** The effects of the intestinal microbiota metabolites or transformed molecules in normal and pathological human conditions (adapted from [42,43]).

Beneficial Microbial Metabolites or Constituents	Advantages	Harmful Microbial Metabolites	Disadvantages
SCFAs	Nutrient, energy providing	Lipopolysaccharide supply	Obesity, metabolic syndrome, leaky gut
Propionate production	Gluconeogenesis, cholesterol lowering	Toxin production	Cancer promotion
Butyrate production	Cancer prevention, colonocyte energy	Tissue invasion of metabolites	Infections, leaky gut
Vitamin productions: B:1,2,5,6,7,8,9,11,12. Vitamin K	Various metabolic cellular effects	Leaky gut induced by metabolites	Autoimmune disease, Inflammatory bowel disease, immune disorders
Anti-inflammatory signals	Normal gut immune function	Microbial enzyme’s PTMP	Autoimmune and allergic disease
Antimicrobial production	Pathogen fighting	Pro-inflammatory signals^3^	Inflammatory bowel disease, immune disorders
Non-digestible carbohydrates-bulk effect	Improved intestinal motility	Acetate production	Hypercholesterolemia, cardiovascular diseases
Bile acids	Improved fat/vitamin absorption, gut barrier, regulate serum lipids and glucose	Secondary bile acids	Colon cancer
Microbial proteases	Protective of intestinal permeability [45]	Microbial proteases	Harmful for intestinal permeability [45]
		Red meat rich L-carnitine metabolism	Atherosclerosis
		Organic acids	Hypertension, obesity, colonic cancer, autism
		Metabolic imbalance	Irritable bowel syndrome, metabolic syndrome
		Amino acids: tyrosine to phenols	Colonic cancer, autism
		Trimethylamine production	coronary vascular disease

**Table 2:** Environmental factors that breach tight junction integrity and increase intestinal permeability. (Adapted from references: [10,12,23,45,60,61,62,65,66,67,68,69]).

Categories	Names	Categories	Names
Pathogens	*H. pylori*	drugs	Proton pump inhibitors
	Enteropathogenic *E. coli*		Non-steroidal anti-inflammatory drugs
	Enterohemorrhagic *E. coli*		Selected bile salts
	*V. parahemolyticus*		
	*Salmonella enterica*/typhimurium	toxins	Clostridium toxin
	*Clostridium difficile*		Ochratoxin A
	*Clostridium perfringens*		Marine toxins
	*Bacteroides fragilis*		EDTA
	*Vibrio cholerae*		
	*Shigella flexneri*	Lifestyle factors	Western diet
	*Campylobacter jejuni*		
	Reovirus		Obesity
	Rotavirus	Gut perfusion	Hypoperfusion
Nutrients	High fat diet	Microbial enzymes	Proteases [45]
	High carbohydrate diet	Allergens	Peanuts, soybean, wheat, milk proteins, nuts, sesame
	Vitamin A deprivation	Carcinogens	Arsenic, phenols, mercury, azoxymethane
	Vitamin D deprivation	Stress	Stress related psychiatric disorders
	Fructose	High-intensity exercise	
	Gluten		
	Processed food additives: sugar, salt, organic acids, microbial transglutaminase, emulsifiers, nanoparticles		
	Medium chain fatty acids		
	Acyl carnitines		

**Table 3:** Environmental factors that enhance TJ integrity and regulate intestinal permeability (Adapted from references: [45,60,61,62,64,70,71]).

Categories	Names
Prebiotic Nutrients	Galactooligosaccharides
	Fructooligosaccharides
Short chain fatty acids	Butyrate
Polyunsaturated fatty acids	PUFA
Nutrients	Glutamine
	Zinc
Plant-derived flavonoids	Quercetin and its metabolites
	Propolis
	Green tea, coffee, berries, grapes, and other fruits/vegetables
Vitamins	A, D
Probiotics	*E. coli nissle 1917*
VSL#3	*Lactobacillus plantarum MB452*
VSL#3	*Bifidobacterium infantis Y1*
	*Lactobacillus salivarius* UCC118
	*Lactobacillus salivarius* CCUG38008
	*Lactobacillus rhamnosus* GG
	*Lactobacillus casei* DN-114 001
	*Lactobacillus casei* Shirota
Microbial enzymes	Proteases [45]
Chemical compounds	Gelatin tannate [71]

**Table 4:** A summary of brain-affecting disorders, where gastrointestinal manifestation exists and the intestinal microbiome and enteric nervous networks are actively involved.

Diseases	Reference
Parkinson’s disease	[84,113,114,115,116,117,118,119,120,121,122,123,124,125,126,127,128]
Autism spectrum disorder	[84,129,130,131,132,133]
Amyotrophic lateral sclerosis	[84,134,135,136]
Alzheimer diseases	[84,137,138,139]
Prion diseases	[81,84,94,139,140,141,142,143,144,145,146,147]
Creutzfeldt-Jakob disease	[81,143,145]
Transmissible spongiform encephalopathies	[84,139,143,145,146]
Additional conditions	
Depression	[148,149,150,151,152]
Anxiety	[150,151,153]
Behavior	[154,155,156]
Cognition	[157,158,159]
Mood	[67,160,161]
Stress	[151,162,163,164]
Fatigue	[165,166,167,168]
Aging	[108,138,169]
